# Severe filarial chyluria Managed successfully with sclerotherapy: A case report from Sudan

**DOI:** 10.1016/j.eucr.2025.102961

**Published:** 2025-01-23

**Authors:** Eltahir Ahmed Eltahir, Ghassan Mahmoud Mohammed Yousuf, Walyeldin Elnour Mohamed Elfakey, Moneer Ali Abdallah, Muna Mohammed Ahmed Hamid Ahmed

**Affiliations:** a University of Elfashir, Darfur, Sudan; b Kampala International University (KIU), Uganda; c Faculty of Medicine, University of Sinnar, Sennar, Sudan; d Department of Pediatrics and Child Health, Faculty of Clinical Medicine and Dentistry, Kampala International University, Uganda; e Department of Pediatrics and Child Health, Faculty of Medicine, University of Bahri, Sudan; f Faculty of Medicine Al Neelain University, Khartoum, Sudan

**Keywords:** Chyluria, Wuchereria bancrofti, Sclerotherapy, Povidone-iodine

## Abstract

**Introduction:**

Chyluria is characterized by the passage of milky urine resulting from abnormal lymphatic drainage into the urinary tract. In parasitic cases, it is commonly caused by Wuchereria bancrofti and has varying severities. Diagnosis relies on clinical, laboratory, and radiological assessments, with treatment options spanning dietary modifications to surgical interventions.

**Case presentation:**

A 43-year-old male presented with chyluria for one year. He received medical treatment for filariasis without response. sclerotherapy with 5 % povidone Iodine was done successfully.

**Conclusion:**

Severe cases of chyluria can be treated successfully with povidone Iodine 5 %.

## Highlights

• Chyluria results from abnormal lymphatic drainage into the urinary tract.
• The patient, after failing medical treatments, underwent successful sclerotherapy without significant complications.
• Treatment: Sclerotherapy with povidone Iodine proved effective for moderate to severe chyluria.
• Conclusion: Sclerotherapy with povidone Iodine is a safe and effective treatment for severe cases of chyluria.

## 1 Introduction

Lymph flows from the intestinal lymphatics to the thoracic duct and subsequently enters the left subclavian vein. Chyluria, characterized by the passage of milky-appearing urine, typically arises from abnormal retrograde or collateral lymphatic flow from the intestinal lymphatics into the lymphatics of the kidney, ureter, and bladder (KUB) system. The condition occurs when lymphatic vessels rupture and drain into the urinary tract. Chyluria was first described by Hippocrates around 400 BCE.^1^ While most reported cases originate from South Asia, a smaller number have been documented in sub-Saharan regions.^2^^,^^3^ Causes of chyluria may be parasitic or non-parasitic. The common cause of parasitic infestation is Wuchereria bancrofti in 95 % of cases.^4^

The most common clinical presentation of chyluria is the passage of milky urine, observed in approximately 70 % of cases. The condition is classified based on severity into mild, moderate, and severe categories.^4^^,^^5^

Intermittent episodes of milky urine characterize mild cases. Moderate cases present with sporadic episodes of milky urine, with or without clot colic. Severe cases involve persistent milky urine accompanied by one or more additional features, such as urinary retention, haematochyluria, or systemic symptoms like weight loss.^5^

Chyluria is suspected clinically and confirmed by laboratory and radiological investigations. Management depends on the severity of the disease and ranges from dietary modification to surgery.^6^ This case report was done following the SCARE guideline for case reports.^7^

**Case report:** A forty-three-year-old Sudanese male from Sennar State southeast Sudan presented to the urology clinic with milky urine for one year mainly at night and early morning, associated with lower urinary tract symptoms. There is no history of hematuria, back pain, trauma, or previous surgeries. However, he did mention that he had lost a significant weight during this time. Clinical examination revealed, a male of 170 cm, weighing 50 kg. Hemodynamically stable, with an unremarkable systemic examination.

**Fig. 1:**
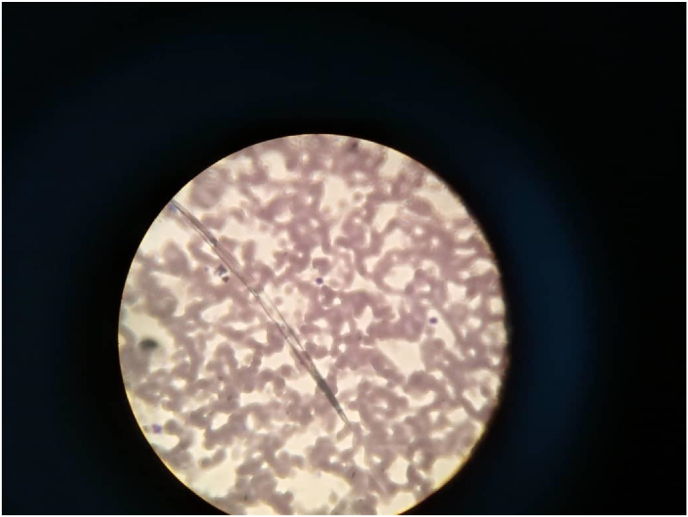
Shows filarial parasites in peripheral blood film.

**Fig. 2:**
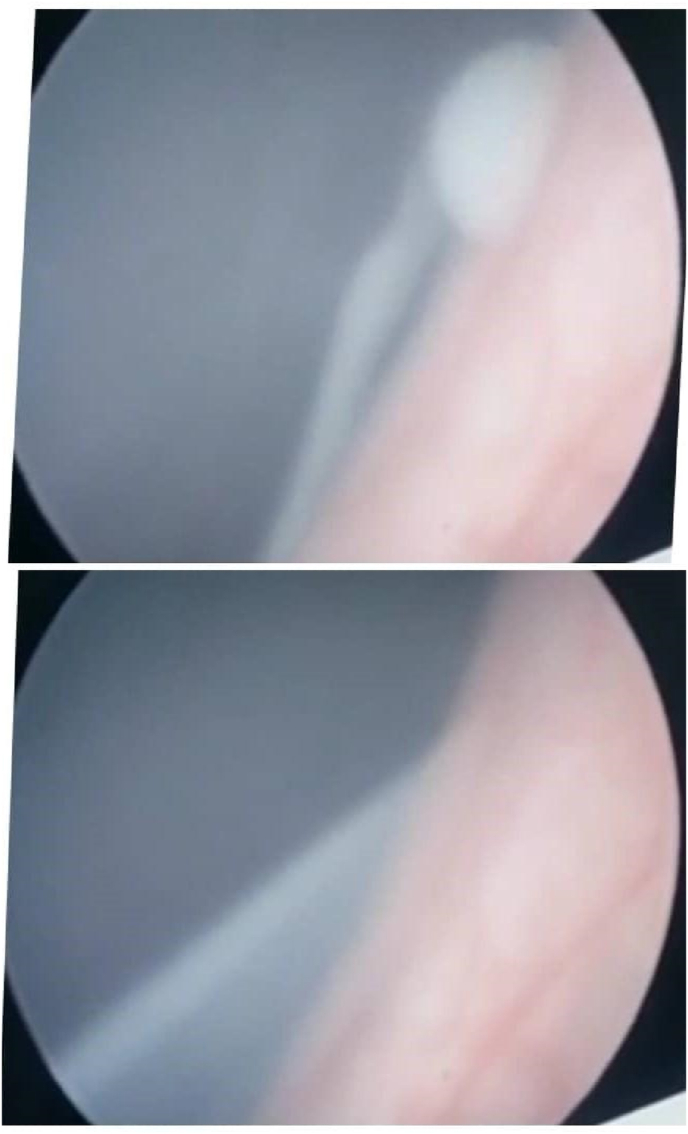
Shows: chyluria coming from the left ureter during cystoscopy.

Urine was milky in color, with two crosses of protein, triglyceride was 180 mg/d. 24 hours urine for Albumin was normal, serum cholesterol was normal, serum Albumin was 3.0 mg/dl, (CBC), kidney function, and liver function tests were all normal. Ether was added to a test tube filled with the cloudy urine rendering the mixture clear suggesting chyluria, A peripheral blood smear confirmed the presence of filarial parasites (Fig. 1). An abdominopelvic ultrasound and chest X-ray showed no abnormalities. Based on these findings, the patient underwent cystoscopy, and a milky white fluid was observed coming from the left ureteric opening during the procedure (Fig. 2).

**Fig. 3:**
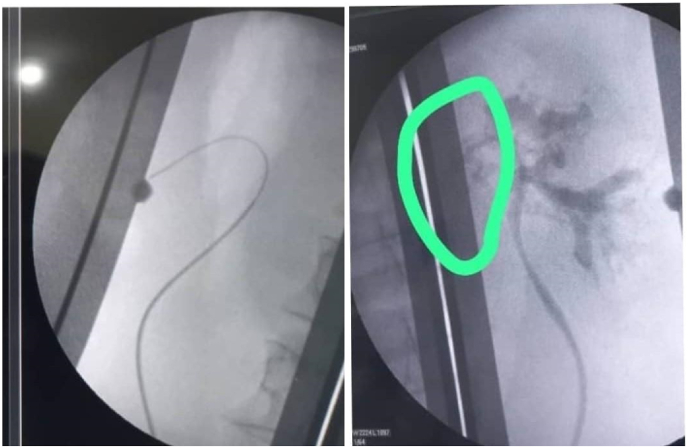
Shows the retrograde pyelogram, that delineated the site of the chylulymphatic fistula.

We started first with a modification of dietary food and, anti-filariasis Ivermectin tablets 200 μg/Kg body weight for six days, this dose was repeated after 3 months, and after six months, but there was no improvement in his main complaint (milky urine). The patient consented to a retrograde pyelogram where a left pyelo-lymphatic fistula was confirmed (Fig. 3). As a result, sclerotherapy was initiated using povidone-iodine. A ureteral catheter was inserted, and a solution of 10 ml of 10 % povidone-iodine mixed with 10 ml of distilled water was prepared (this equals 20 ml of 5 % povidone Iodine). The solution was injected in a 10 ml syringe through the ureteral catheter, held for 5 minutes, and then released. This process was repeated until the full amount of the solution was administered. The treatment was administered three times daily for three consecutive days.

The patient was also treated with a third-generation cephalosporin (1 g twice daily) for three days of sclerotherapy and discharged on oral antibiotics Amoxicillin + clavulanic acid (1 gm twice daily for seven days) following the removal of the ureteral stent. There were no significant adverse effects during or after the procedure. The patient was seen two weeks following surgery and then monthly for one year. No recurrence of chyluria was observed during the follow-up period, and the patient reported no significant complications.

## 2 Discussion

Chyluria, characterized by the passage of milky urine, occurs due to abnormal retrograde or collateral lymphatic flow into the urinary tract, typically resulting from ruptured lymphatic vessels.^3^

The patient's presentation aligns with a severe classification of chyluria. Persistent milky urine and weight loss are hallmark features of severe cases, though the absence of urinary retention or haemato-chyluria indicates a slightly less severe spectrum^5^

The presence of chyle in urine can be confirmed through a simple sedimentation test. This is done when a urine sample is left to settle in a test tube, and it separates into three distinct layers: a fatty upper layer, a fibrinous middle layer, and sediment debris at the bottom^8^ Using dark-field microscopy, chylomicrons can be identified, and these can be further stained with Sudan III for visualization.^9^ In resource-limited settings, the application of ether, as performed in this can effectively clear the fatty top layer and serve as an appropriate alternative. Quantitative analysis of urinary triglycerides can also be performed using a biochemical analyzer or photoelectric calorimeter for more precise measurements.^5^ In this case, the detection of chyluria was done physically by observing the urine sample while sediments in the test tube and under microscopy by visualizing lymphocytes. *Wuchereria bancrofti* causes about 95 % of obstructive chyluria. In Asia, lymphatic filariasis can also be caused by Brugia malayi and Brugia timori.^4^ In this case, microfilaria of *Wuchereria bancrofti* was detected in a peripheral blood smear (Fig. 1). Cystoscopy helps determine the side involved, in this case, it is the left side (Fig. 2).

Since the underlying cause was proved to be filariasis, given the fact that the patient is from an endemic area in Sudan, we started first with modification of dietary food and anti-filariasis Ivermectin tablets 200 μg/Kg body weight for six days, repeated after three months and six months but the patient complained did not improve. The failure of medical treatment in this patient can be explained by the severity of the case, evident by the intermittent passage of milky urine and loss of weight.^5^^,^^6^

Goyal NK et al. have studied 222 patients. Thirty-one patients did not respond to conservative management, and treatment failure was associated with more severe disease, a higher number of prior medical treatment courses, and greater baseline cholesterol loss compared to responders. The success rate was seventy per cent (70 %).^6^ when conservative treatment fails, the installation of sclerosant material in the renal pelvis is carried out. When lymphatics heal by fibrosis, chyluria stops immediately. Different sclerosants are in use nowadays the commonest among them is povidone Iodine. We used it in a concentration of 5 %. The procedure was done successfully with no post-operative complications, this is our first experience with this procedure. We selected povidone Iodine because it is available, affordable, and safe.

Sinha RK reported the use of povidone Iodine in a concentration of 0.2 %–5 %. Without adverse effects, and a success rate of about 90 %.^3^

Silver nitrate (0.1–1%); Sabnis RB et al. studied sixty-two patients with chyluria for installation with silver nitrate 1 % in the renal pelvis, with a resultant success rate of 82 %.^10^ In cases where sclerotherapy is not successful surgical treatment is advised. These include chylo-lymphatic disconnection, lymph-venous anastomosis, retroperitoneal lymph-venous anastomosis, trans inguinal spermatic lymph-venous anastomosis, inguinal lymph node-saphenous vein anastomosis, and more definitive approaches such as auto transplantation or nephrectomy.^3^

## 3 Conclusion

This case report shows that severe cases of chyluria can be effectively treated with sclerotherapy using Povidone Iodine. It is safe, accessible, and effective in the treatment of refractory chyluria. Future studies should focus on optimizing treatment protocols and evaluating long-term outcomes in larger patient populations.

## Research registration

Not applicable.

## Provenance and peer review

Not commissioned, externally peer-reviewed.

## CRediT authorship contribution statement

**Eltahir Ahmed Eltahir:** Conceptualization, Data curation, Writing – review & editing. **Ghassan Mahmoud Mohammed Yousuf:** Conceptualization, Data curation, Investigation, Writing – original draft. **Walyeldin Elnour Mohamed Elfakey:** Writing – review & editing. **Moneer Ali Abdallah:** Writing – review & editing. **Muna Mohammed Ahmed Hamid Ahmed:** Writing – review & editing.

## Limitations of this study

This case report represents a single patient and therefore, the results cannot be generalized. Additionally, the study was conducted in a resource-limited setting where certain investigations, such as lymphoscintigraphy, were not available.

## Author agreement statement

1) We the undersigned declare that this manuscript is original, has not been published before, and is not currently being considered for publication elsewhere.

2) We confirm that we have read and approved the manuscript and are the only persons responsible for its authorship.

3) We understand that the Corresponding Author is the sole contact for the Editorial process. He is responsible for communicating with the other authors about progress and final approval of proofs.

4) We transfer the copy to the Journal of Interventional Medicine.

## Consent for publication

Written informed consent was obtained from the patient for publication of this case report and accompanying images. A copy of the written consent is available for review by the Editor-in-Chief of this journal on request.

## Ethical approval

Ethical approval was obtained from the local ethical committee/research, ethical local committee, Al Neelain University; Khartoum Sudan.

## Financial Support

No funding was received for this study.

## Conflicts of interest

The authors declare no conflicts of interest.
